# The effect of resveratrol on oxidative stress in the liver and serum of a rat model of polycystic ovary syndrome: An experimental study

**Published:** 2018-03

**Authors:** Mahnaz Ghowsi, Homayoun Khazali, Sajjad Sisakhtnezhad

**Affiliations:** 1 *Department of Animal Physiology, Faculty of Biological Sciences and Technology, Shahid Beheshti University, Tehran, Iran.*; 2 *Department of Biology, Faculty of Sciences, Razi University, Kermanshah, Iran.*

**Keywords:** Oxidative stress, Polycystic ovary syndrome, Resveratrol, Liver

## Abstract

**Background:**

Studies of oxidative status in polycystic ovarian syndrome (PCOS) patients are limited with inconsistent results. The effects of resveratrol as a natural antioxidant on oxidative status in PCOS aren’t clear.

**Objective:**

This study evaluated effects of resveratrol on oxidative stress in the liver and serum of the PCOS rats.

**Materials and Methods:**

Fifteen female *Wistar* rats (3 wk old) were divided into 3 groups (n=5/each e): Control group, PCO-Control group, and PCO-Resveratrol group. For induction of polycystic ovary phenotype, testosterone enanthate 10 mg/kg was injected for 35 days subcutaneously. Then, resveratrol 10 mg/kg was injected intraperitoneally for 28 days to rats of the PCO-Resveratrol group. Ovarian sections were stained with hematoxylin/eosin. The serum glucose and insulin and the levels of malondialdehyde (MDA) and total antioxidant capacity (TAC) in serum and liver were measured.

**Results:**

Control animals showed normal ovarian morphology and PCO-Control animals exhibited cystic follicles. There were no significant differences in liver TAC between groups. The serum MDA (p=0.034), and homeostatic model assessment insulin resistance (HOMA-IR) (p=0.014) levels in PCO-Control rats were higher than the controls. The liver MDA in PCO-Control rats was more than that of controls (p=0.001). The HOMA-IR (p=0.008) and serum MDA (p=0.006) levels in PCO-Control rats were more than those of PCO-Resveratrol rats (p=0.008). In PCO-Resveratrol group, serum TAC was higher than that of PCO-Control group (p=0.022) and liver MDA was more than controls (p=0.01).

**Conclusion:**

Results indicated that the induction of PCOS in rats increased lipid peroxidation and insulin resistance and resveratrol improved these complications.

## Introduction

Polycystic ovary syndrome (PCOS) is an endocrine and metabolic disorder that affects 6-14% of women in childbearing age. The incidence of obesity, insulin resistance, and risks for type 2 diabetes mellitus and cardiovascular disease are high among PCOS women. Some evidence suggests that oxidative stress may play an important role in infertility and heterogeneous disorders that are seen in many PCOS patients (1). Oxidative stress is defined as an imbalance between oxidants and antioxidants in the living organisms. Many studies have shown that oxidative stress is generally present in the women with PCOS without considering that they are lean or they have metabolic disorders (2). The basic mechanisms for oxidative stress in PCOS are not completely understood, yet new researches powerfully imply that insulin resistance plays an axial role in the pathogenesis of PCOS and it augments oxidative stress. In oxidative stress, free radicals, production of highly toxic products such as malondialdehyde (MDA), and other lipid peroxidation products may cause insulin resistance (3). The results of the evaluation of oxidative stress in the women with PCOS are conflicted; for example, two studies have been reported an increase in the total oxidant status in PCOS patients, comparisone to control subjects (4). But, no differences were shown in another study (4). Also, serum lipid peroxide (lipid oxidation products) concentrations were increased in patients with PCOS in another study (5). However, in another research, plasma thiobarbituric acid-reactive substances levels, that are byproducts of lipid peroxidation, were similar in the PCOS patients and control subjects (6). One organ that may be attacked by reactive oxygen species (ROS) is liver, but studies about the evaluation of oxidative stress in liver of PCOS patients are limited. 

Resveratrol (3, 4′, 5-trihydroxy-trans-stilbene) is a phytochemical with anti-carcinogenic, anti-inflammatory, antioxidant and cardioprotective effects which is found in some dietary sources such as grapes, berries, peanuts, and red wine (7). Some studies have shown that it has glucose lowering effects (8). In addition, the antioxidant effect of resveratrol in preventing lipid oxidation and scavenging of ROS is strong (7). Some studies have suggested that resveratrol has poor direct antioxidant effects and its protective effects may be via an increase in the activity of cellular antioxidant enzymes including superoxide dismutase, catalase, and glutathione peroxidase (9). However, it has been shown that resveratrol may not be useful in some rodent models (7). 

Due to conflicting results of some studies that have addressed oxidative stress and PCOS (4), this study was designed to evaluate the levels of malondialdehyde and total antioxidant capacity (TAC) as two biomarkers of oxidative stress in liver and serum of a rat model of PCOS. Moreover, we evaluated the effects of resveratrol as an important antioxidant and glucose lowering agent on these parameters because the improvement of oxidative status may be helpful in reduction of metabolic complications associated with PCOS. 

## Materials and methods

**Experimental design and animals**

In this study, all experiments were carried out on immature female Wistar rats (n=15, age 21 days). All efforts were made to minimize the number of animals that were used. The animals were purchased from Shahid Beheshti University (Tehran, Iran), housed in a well-controlled conditions (at 23±2^o^C, relative humidity of 50±5%, 12 hr light/dark cycle). The rats had free access to food and water *ad *libitum. 

**Figure 1:**
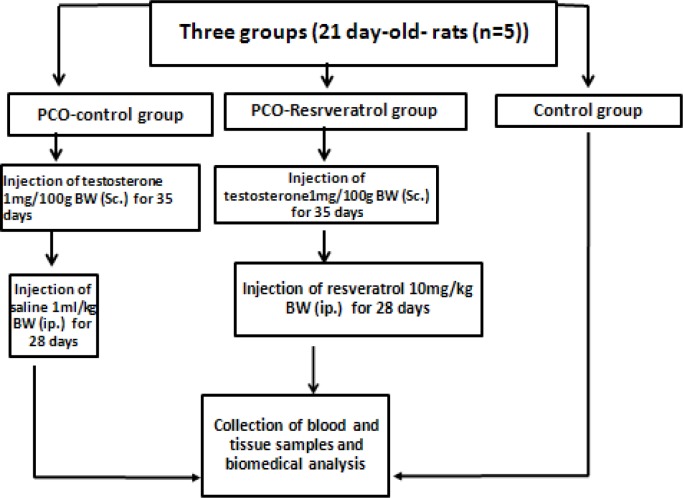
The experimental protocol. The abbreviations denote the following: Control group: these rats had no treatment; PCO-Control group: these rats received testosterone enanthate 1 mg/100 g body weight subcutaneously once daily for 35 days. Then, they received saline for 4 wk intraperitoneally; PCO-Resveratrol group: these rats received testosterone enanthate 1 mg/100 g body weight subcutaneously once daily for 35 days. Then, they received resveratrol intraperitoneally for 4 wk once daily.

The animals were randomly divided into three groups as follows: 1) The control group (n=5 served as control rats and had no treatment 2). The PCO-Control group (n=5) received testosterone enanthate 1 mg/100 g body weight (dissolved in sesame oil) subcutaneously once daily for 35 days. This group was used as a model of PCOS (10). Then, the animals of this group were treated with 1 mL/kg/day isotonic saline intraperitoneally for 4 wks, and 3). The PCO-Resveratrol group (n=5) served as the PCOS induced rats that received testosterone enanthate 1 mg/100 g body weight (dissolved in sesame oil) subcutaneously once daily for 35 days. Then they were treated with resveratrol 10 mg/kg body weight (Figure 1). Resveratrol (Sigma Aldrich, Missouri) was injected intraperitoneally for 4 wks once daily (11).

**Experimental investigation**

At the end of the treatments, a combination of ketamine hydrochloride (50 mg/kg) and xylazine hydrochloride (7 mg/kg) was injected to the overnight fasted rats intraperitoneally and they were anesthetized. The body weight and the body lengths (nasal-anal length) of rats were measured. Then, the Lee’s index (=body weight1/3 (g)/ nasoanal length (cm) × 1000) was estimated. Blood samples were collected by cardiac puncture and were placed into tubes and centrifuged. The obtained serum of each rat was kept at -30^o^C until use. According to the TAC manufacturer’s instruction of assay kit (ZellBio GmbH, Wurttemberg, Germany) instruction, the liver samples were taken and weighed up. 200 µl of phosphate buffer saline (pH=7.4, 100 mM) was added and frozen in liquid nitrogen immediately for later use. Then the liver samples were thawed and were kept in 2-8^o^C. In the next stage, 800µl of phosphate buffer saline (pH=7.4) was added to them. Then, the samples were thoroughly homogenized and centrifuged (at 4000 rpm for approximately 20 min). Finally, the supernatants were collected carefully, aliquoted, and kept at -20^o^C for later use. 

**Ovarian morphology**

The ovaries from the rats in the control group and the PCO-control group were removed and fixed in 4% neutral buffered formalin (pH=7.4) for 48 hr at room temperature. The samples were dehydrated, processed in paraffin blocks and sectioned at 5  μm for histological analyses. The hematoxylin and eosin staining were done and slides were assayed. 

**Measurement of TAC levels in serum and liver**

The TAC levels in serum and liver tissue were measured by using a commercial kit (ZellBio GmbH, Wurttemberg, Germany) available for the quantitative assay of the antioxidant capacity on the basis of the oxidation-reduction colorimetric assay. The TAC amounts in the sample from each animal were determined according to the manufacturer’s instructions colorimetrically at 490 nm. The detection range was 0.125-2 mM (125-2000 µmol/L). The intra- and inter-assay coefficients of variation were <3.4% and <4.2% respectively. The sensitivity of the kit was 0.1m M (100 µmol/L). In this assay, TAC amount is considered as the amount of antioxidant in the sample that will compare with ascorbic acid that acts as a standard.

**Determination of MDA in serum and liver samples**

An analytical grade of MDA, methanol, 2-thiobarbituric acid (TBA), Butylated hydroxyl-toluene (BHT), and 1, 1, 3, 3- tetraethoxy-propane (TEP) used in this study were from Sigma (Sigma-Aldrich Co., St Louis, MO, USA). The other substances were purchased from Merck (Merck, Darmstadt, Germany). An Agilent Technologies 1200 Series HPLC system (Agilent Corp., Germany) with EC 250/4.6 Nucleodur 100–5C18ec column (Macherey-Nagel, Duren, Germany) was used according to following protocol to measure MDA levels in serum and liver homogenate samples. Each sample of serum or liver homogenate or TEP standard (50 µL of a stock standard solution containing 5 µmol L-1 TEP in 40% ethanol solution) was added to 50µL BHT (0.05% v/v BHT in ethanol), 400µL H_3_PO_4_ and 100 µL TBA (42 mmol L-1 in 0.44 mol L-1 H_3_PO_4_) and keep warm at 100^o^C for 1 hr. Then, samples were chilled for 10 min. Then, the MDA–TBA complex was removed from the mixture with n-butanol (250 µL). 

The samples were mixed for 5 min and centrifuged for 3 min at 14000 g to separate the two phases. Aliquots of 100 µL were extracted from an n-butanol layer of each sample. For measurement of MDA, 20 µL of the n-butanol extract was injected into an HPLC reverse-phase column using a mixture of methanol and 50 mmol L–1 phosphate buffer, pH=6.7 (40/60, v/v) as the mobile phase. The MDA peak at 553 nm, excitation 515 nm was detected. To confirm the values of the eluted MDA, a commercial standard was used and its quantity was determined by peak area using Agilent Technologies 1200 Series software (12).

**Determination of insulin hormone and glucose concentrations in serum samples**

The glucose concentrations in the serum samples were measured by a commercial kit (zist, chemistry, Iran) upon GOD-PAP/Endpoint method. In addition, the insulin concentrations of serum samples were determined using a Mercodia Rat insulin ELISA kit (Mercodia AB, Sylveniusgatan 8A, SE-754 50 Uppsala, Sweden). The coefficient of variation within and between assays was 3.1% and was 4.4% respectively.

**Ethical consideration**

Animal usage and the protocols were approved by the Institutional Animal Care and Ethical Committee of Biological Sciences of Razi University (no: 396-2-017).

**Statistical analysis**

All data are shown as the mean±SEM. The data were analyzed by one-way analysis of variance (ANOVA) using SPSS (Statistical Package for the Social Sciences, version 16.0, SPSS Inc, Chicago, Illinois, USA) software, followed by Tukey’s test analysis to discriminate the main effects and to compare various groups with each other. p<0.05 were considered statistically significant.

## Results

**Ovarian morphology**

**Figure 2:**
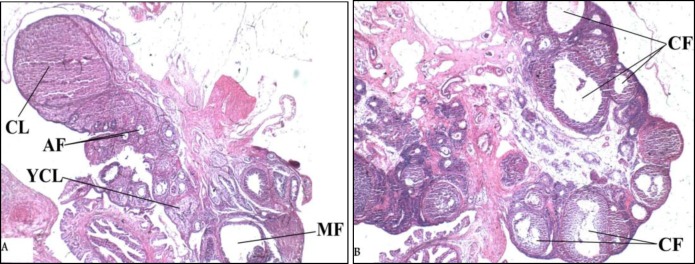
Photomicrographs of sections of the ovaries stained by Hematoxylin-Eosin. (×40 magnifications). (A) The section from the Control group that shows normal ovarian morphology (B). The section from the PCO-Control group that displays a considerable number of cystic follicles. The abbreviations denote the following: CL: Corpus luteum, YCL: Young corpus luteum. AF: Antral follicle, MF: Mature follicle, CF: Cystic follicle.

In the ovary slides of the control group, the corpora lutea and small and medium-sized antral follicles were seen (Figure 2A). The cystic follicles and atretic follicles with no corpora lutea were seen in the ovary slides of polycystic ovarian rats (Figure 2B). These results were similar to features of PCOS model that has been introduced by Beloosesky and colleagues (10). Thus signs of PCOS in rat model were established, but due to the aim of this study, the data related to the counting of the signs isn’t shown here.

**Determination of the body weight and Lee’s index**

The mean of final body weights of rats in the PCO-Resveratrol group was statistically higher than the mean of weights in the PCO-Control group (Table I) (p=0.048). In the PCO-Control group, the mean of Lee’s index values was statistically higher compared to the control group (Table I) (p=0.014), but in the PCO-Resveratrol group, the mean of Lee’s index was not statistically different compared to the PCO-Control group (p=0.066) and Control group (p=0.663) (Table I).

**Determination of TAC and MDA levels in the samples of serum and liver tissue**

**Figure 3:**
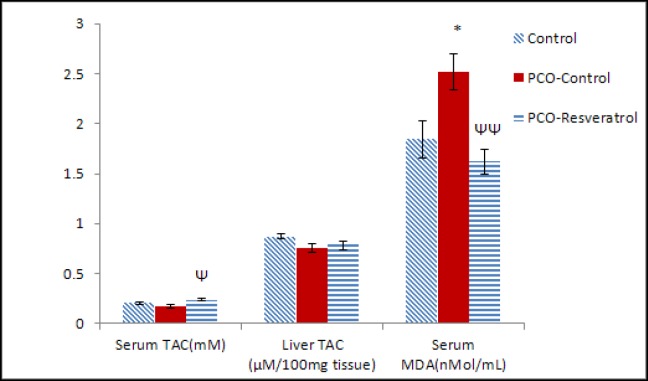
The fasting levels of TAC and MDA in the serum and the levels of TAC in the liver tissue of different groups at the end of treatments. Each group had 5 rats. TAC amount was considered as the amount of antioxidant in the sample that was compared with ascorbic acid that acts as a standard. The data are given as Mean±SEM.

The mean of serum TAC levels in the PCO-Resveratrol group was statistically higher than the mean of serum TAC in the PCO-Control group (p=0.022) (Figure 3). In the PCO-Control group, the serum TAC levels were not statistically different from serum TAC levels in the control group (Figure 3). The TAC levels in the liver tissues of the PCO-Control group were not statistically different from those of the Control group (p=0.063). The mean of TAC levels in the PCO-Resveratrol group was statistically similar to the PCO-Control group (p=0.743) (Figure 3).

**Figure 4:**
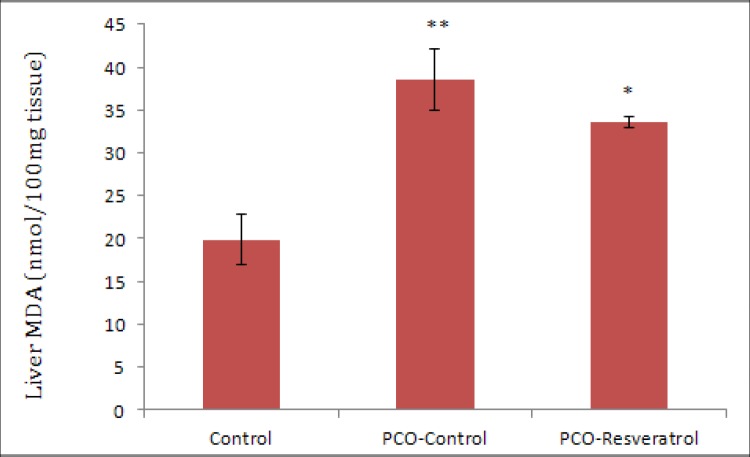
The fasting levels of MDA in the liver tissue of different groups at the end of the study. Each group had 5 rats. The data are given as Mean±SEM.

The results of this study showed that the mean of serum MDA levels of the PCO-Control rats was increased in comparison with the serum MDA levels in the control rats (p=0.034). Furthermore, the mean of serum MDA concentrations in the PCO-Resveratrol group was significantly lower than that of the PCO-Control group (p=0.006) (Figure 3). The mean of MDA levels in the livers of the PCO-Control group was higher than the MDA levels of control rats (p=0.001) and the mean of MDA levels in the livers of the PCO-Resveratrol group was lower than that of the PCO-Control group, but not significantly (p=0.423) (Figure 4).The mean of MDA levels in the livers of the PCO-Resveratrol group was higher than the MDA levels in thecontrol group rats (p=0.01) (Figure 4).

**Determination of insulin, glucose, and HOMA insulin resistance (HOMA-IR) levels in the serum samples**

**Figure 5:**
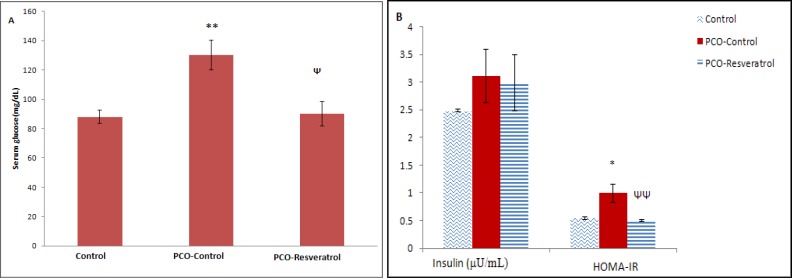
The fasting serum glucose levels (mg/dL) (A); the fasting serum insulin levels (μU/mL) and the HOMA-IR index (B) in the different groups at the end of the study. Each group had 5 rats. The data are given as Mean±SEM.

The mean of fasting serum glucose levels of the PCO-Control group was statistically higher than that in the Control group (p=0.008). In addition, the mean of concentrations of serum glucose in the PCO-Resveratrol group was significantly lower than the mean of glucose concentrations in the PCO-Control group (p=0.011) (Figure 5A). The mean of fasting serum insulin levels was not statistically different between various groups (p<0.05) (Figure 5B). In this study the homeostatic model assessment insulin resistance (HOMA-IR) levels were calculated according to Aref and colleagues, 2013 as follows:

HOMA-IR=fasting insulin × fasting glucose/405 (13).

Our findings indicated that the mean of HOMA-IR levels in the animals of the PCO-Control group was increased compared to the Control group (p=0.014). The mean of HOMA-IR levels in the PCO-Resveratrol group was significantly lower than that in the PCO-Control group (p=0.008) (Figure 5B).

**Table I:** Determination of the body weight and Lee’s index

**Groups**	**Body weight (g)**	**Lee’s index**
Control	204.8 ± 5.044	325.4 ± 1.995
PCO-Control	205.8 ± 9.124	349.1 ± 6.218*
PCO-Resveratrol	224.2 ± 5.380^Ψ^	331.6 ± 5.523

Data presented as mean ± SEM. Each group had 5 rats.

* Significantly different from the Control group (p< 0.05)

Ψ significantly different from in the PCO-Control group (p<0.05) (One-way analysis of variance followed by Tukey’s test).

*Significantly different from the Control group (p<0.05)

Ψ Significantly different from the PCO-Control group (p<0.05)

Ψ Ψ Significantly different from the PCO-Control group (p<0.01)

*Significantly different from the Control group (p<0.05)

**Significantly different from the Control group (p<0.01)

**Significantly different from the Control group (p<0.01)

Ψ Significantly different from the PCO-Control group (p<0.05)

* Significantly different from the Control group (p<0.05)

ΨΨ Significantly different from the PCO-Control group (p<0.01).

## Discussion

In the present study, we have evaluated the effects of trans-resveratrol, as an important antioxidant agent, on the oxidative stress biomarkers in a rat model of PCOS. In this model, several important aspects of PCOS complications such as insulin resistance and obesity were been approached. The results of the histological examination of the ovaries revealed significant changes in the ovary tissue of the rats in the PCO-Control group compared to the rats of the Control group. It showed that testosterone enanthate injection caused the development of cystic follicles. The numbers of atretic follicles in the PCO-Control group was increased and no corpus luteum was seen. 

The results of serum glucose and HOMA-IR measurements indicated that animals in the PCO-Control group had insulin resistance. These results are similar to other studies that have indicated that insulin resistance is frequently seen among PCOS patients. It has been shown that insulin resistance and subsequent hyperinsulinemia play important roles in metabolic complications that are associated with PCOS. (14). The present findings of HOMA-IR levels in the PCO-Resveratrol group are in accordance with results of one study that showed that resveratrol possesses hypoglycemic activity in the diabetic rats and they reported that the hypoglycemic activity of resveratrol is not due to the stimulation of insulin secretion (8, 15). Similarly, our study, our results suggest that resveratrol didn’t affect insulin secretion. It has been suggested that the beneficial effects of resveratrol may be due to reducing the blood glucose release by the liver or may be due to increase in the use of glucose by the tissues through potentiation of insulin signalling or its direct action (16). However, further researches are needed to elucidate the exact mechanisms of the effects of resveratrol. Similarly, some previous studies suggested that antioxidant usage improves insulin function in the diabetic patients (1, 4). 

MDA is a biomarker of lipid peroxidation and indicates the level of free radicals that may damagedifferent organs in oxidative stress. In the present study, the mean of serum MDA levels in the PCO-Control group was higher than that in the control rats. There is some evidence that indicates the markers of oxidative stress and inflammation are connected to circulating androgens. In this regard, the excess levels of androgen may induce oxidative stress independently from obeseness. It has been suggested that hyperandrogenism may cause pre-activation of mononuclear cells in the PCOS patients and thereby it may cause an increase in ROS production independently from obesity (1). However, the mechanisms involved in ROS production in PCOS are not known; some studies reported that nicotinamide adenine dinucleotide phosphate oxidase may be involved in the production of ROS in different cells (17). 

In the present study, the serum levels of TAC in the polycystic ovarian rats were not significantly different from the TAC levels in the control rats. The serum TAC is the capability of serum to extinguish free radicals production. TAC helps protection of cells against molecular damages (18). Fenkci and colleagues reported that TAC values in women with PCOS were lower than the age- and BMI-matched controls and in their study the fasting insulin concentrations and TAC values had a negative correlation and they suggested that insulin resistance may alter the antioxidant system in PCOS (19). However, our findings aren’t consistent with this study. Our results about TAC levels are in accordance with the results of a study by Desai and colleagues that showed the ferric reducing ability of plasma levels, an index of TAC, were not significantly different between the non-obese PCOS women and controls (20).

In addition, our findings are inconsistent with results of another study by Fenkci and co-workers that has reported that the TAC levels in none obese PCOS patients with normal insulin were higher than the TAC levels in age- and BMI-matched controls and in that study, they suggested that the increase in TAC level was a compensatory response to oxidative stress (19). 

In the present study, the results about MDA levels in the PCO-Control rats are in accordance with findings of one study by Fenkci and colleagues that reported serum MDA levels were increased in the non-obese PCOS women compared to controls (19). Our findings suggest that there is oxidative stress status in the rats with testosterone-induced polycystic ovaries. Some studies supported that insulin resistance may play a role in PCOS pathogenesis and may augment oxidative stress (1) and results of the present study about HOMA-IR and biomarkers of oxidative stress approved them. Hyperglycemia may stimulate the production of tumor necrosis factor-α from mononuclear cells that may result in inflammation and insulin resistance. Chronic hyperglycemia may result in oxidative stress by different mechanisms, for example, changing the redox balance via the increase in polyol pathway flux, overproduction of superoxide in the mitochondrial electron transport chain, and direct production of ROS (16). In addition, it has been demonstrated that ROS production and oxidative stress cause impairment in glucose uptake by muscles and adipose tissue; and may reduce insulin secretion by β-cells of the pancreas (21). ROS cause oxidative damage to a large number of biomolecules and may result in their dysfunction. It has reported that there is a significantly negative correlation between MDA levels and insulin sensitivity. Also, there is a significantly negative correlation between MDA levels and glutathione, an antioxidant, levels (2). These findings may indirectly suggest that insulin resistance may decrease antioxidant levels and it may increase lipid hydroperoxide generation. 

In this study, the mean of serum TAC levels in the PCO-Resveratrol group was higher than that in the PCO-control group. In addition, the mean of serum MDA levels in the animals of PCO-Resveratrol group was decreased in comparison with rats of the PCO-Control group. It has been reported that early treatment with resveratrol in the hepatocytes which were cultured and were exposed to oxidative stress, caused an increase in the antioxidant enzymes activity by the act on the Nrf2 transcription factor (22). Previous studies have shown that resveratrol decreased oxidative stress in rats with streptozocin-induced diabetes (23, 24). Our results are consistent with these studies. It has also been shown that resveratrol decreased the production of free radicals (7). Another mechanism that has suggested for resveratrol effect is its natural antioxidant capacity because its hydroxyl phenolic groups have redox properties (11).

We also investigated TAC and MDA levels in the liver tissue of female rats with testosterone-induced polycystic ovaries. Our results demonstrated that there were no significant differences in the mean of TAC levels in the liver tissues of the various groups. The MDA levels in the livers of polycystic ovarian animals were higher than the MDA levels in livers of control rats. In addition, the mean of MDA levels in livers of PCOS-Resveratrol group was lower than that of PCOS-Control group, but there was not a significant difference. These results suggest an increase in lipid peroxidation and oxidative stress in the livers of animals with polycystic ovaries and they indicated that treatment with resveratrol reduced these effects relatively. The excessive ROS resulting from oxidative stress have critical acts in the liver diseases and many chronic disorders. Oxidative stress initiates hepatic damage by changing of lipids, proteins, and DNA contents and by the alteration in the pathways that control the transcription of the gene, the expression of proteins, cell apoptosis, and hepatic stellate cell activation; thus, it may cause progression of various liver diseases (25). 

Oxidative stress may increase production of cytotoxic pro-inflammatory cytokine TNF. As a consequence, it may stimulate TNF receptors on the cell surface; as a result, it may cause activation of the stress-related protein kinases c-Jun N-terminal kinase (JNK) and IĸK (IĸB kinase) which may lead to the production of additional inflammatory cytokines and result in reduction of insulin sensitivity, accordingly (26). The reduction of the mean of MDA levels in the livers of the PCO-Resveratrol rats and increment of serum TAC levels in PCOS-Resveratrol group may be due to the enhanced activation of Nrf2 by resveratrol. These findings suggest that resveratrol may have hepatoprotective effects on oxidative stress in PCOS, as it has been shown that the activation of Nrf2 by pharmacologic molecules or genetic engineering protects the liver in different oxidative stress models (27). 

The mean of TAC levels in the liver tissues of various groups in the present study wasn’t significantly different. It may be due to the insufficient dose of resveratrol in our study because the results of another study in the diabetic rat livers showed that treating the diabetic rats by 20 mg/kg resveratrol increased the activity of glutathione peroxidase. Furthermore, it was associated with an increase in phosphorylated glutathione peroxidase levels and resveratrol activated a coordinated cytoprotective response against the changes that were induced by diabetes in liver tissue (28). Therefore, we suggest that more studies are needed to confirm that the effects of resveratrol on oxidative stress are dose-dependent or not.

In the present study, in order to estimate the obesity status, we measured Lee’s index in rats at the end of the experimental period. This biomarker is equivalent to body mass index (BMI) in humans (29) and is used as an indicator of body fat excess (30). The mean of Lee’s index values in the PCO-control group was more than the levels of this parameter in the Control group. Also, animals of this group had higher oxidative stress as evidenced by an increase in MDA levels. These results are consistent with a previous study that reported oxidative stress was related to the obesity that commonly seen in PCOS women (31). However, some studies indicated that there is an increase in oxidative stress biomarkers even in non-obese PCOS women (20). Considering the Lee’s index results, adiposity was increased in the PCO-Control group compared to the control rats. In accordance with our findings, other studies have confirmed that obesity is often present in women with PCOS ranging from 30% to 60% (32, 33). 

It has also demonstrated that there are correlations between biomarkers or end-products of free radical-mediated oxidative stress and BMI. In addition, it has found that in individuals with overweight, the antioxidant defense markers had negative correlations with visceral obesity and body fat (34). The accumulation of fat in the obese individuals causes ROS secretion into the circulating blood and it is connected with the initiation of insulin resistance in skeletal muscles and adipose tissues (35). Moreover, there is some evidence that indicates free fatty acids provide the link between obesity and insulin resistance/type 2 diabetes and the free fatty acids may impair insulin-stimulated glucose uptake (36). 

Considering Lee’s index results, adiposity wasn’t decreased in the animals of the PCO-Resveratrol group compared to the PCO-Control group, but previous studies in animals and human have demonstrated that effects of resveratrol are similar to calorie restriction, therefore; further researches are needed to investigate the effect of resveratrol on the obesity status in the PCOS patients. 

## Conclusion

Our study showed that induction of PCOS in immature rats affected the oxidative stress biomarkers in the liver and serum of the animals. In addition, it may cause impairment in the physiological actions of insulin and treatment with resveratrol can improve these effects. Therefore, taken together the results of this study suggest that resveratrol may be an appropriate therapeutic agent for the improvement of metabolic disorders associated with polycystic ovary syndrome.

## Note

***This article extracted from Ph.D. Thesis. (Mahnaz Ghowsi)***
